# Comparison of Gene Expression by Sheep and Human Blood Stimulated with the TLR4 Agonists Lipopolysaccharide and Monophosphoryl Lipid A

**DOI:** 10.1371/journal.pone.0144345

**Published:** 2015-12-07

**Authors:** Perenlei Enkhbaatar, Christina Nelson, John R. Salsbury, Joseph R. Carmical, Karen E. O. Torres, David Herndon, Donald S. Prough, Liming Luan, Edward R. Sherwood

**Affiliations:** 1 Department of Anesthesiology, the University of Texas Medical Branch, Galveston, TX, United States of America; 2 Molecular Virology and Microbiology Core, Alkek Center for Metagenomics & Microbiome Research, Baylor College of Medicine, Houston, TX, United States of America; 3 GenUs BioSystems, Northbrook, IL, United States of America; 4 Shriners Hospital for Children, Galveston, TX, United States of America; 5 Department of Anesthesiology, Vanderbilt University Medical Center, Nashville, TN, United States of America; University of Ulm, GERMANY

## Abstract

**Background:**

Animal models that mimic human biology are important for successful translation of basic science discoveries into the clinical practice. Recent studies in rodents have demonstrated the efficacy of TLR4 agonists as immunomodulators in models of infection. However, rodent models have been criticized for not mimicking important characteristics of the human immune response to microbial products. The goal of this study was to compare genomic responses of human and sheep blood to the TLR4 agonists lipopolysaccharide (LPS) and monophosphoryl lipid A (MPLA).

**Methods:**

Venous blood, withdrawn from six healthy human adult volunteers (~ 28 years old) and six healthy adult female sheep (~3 years old), was mixed with 30 μL of PBS, LPS (1μg/mL) or MPLA (10μg/mL) and incubated at room temperature for 90 minutes on a rolling rocker. After incubation, 2.5 mL of blood was transferred to Paxgene Blood RNA tubes. Gene expression analysis was performed using an Agilent Bioanalyzer with the RNA6000 Nano Lab Chip. Agilent gene expression microarrays were scanned with a G2565 Microarray Scanner. Differentially expressed genes were identified.

**Results:**

11,431 human and 4,992 sheep probes were detected above background. Among them 1,029 human and 175 sheep genes were differentially expressed at a stringency of 1.5-fold change (p<0.05). Of the 175 sheep genes, 54 had a known human orthologue. Among those genes, 22 had > 1.5-fold changes in human samples. Genes of major inflammatory mediators, such as IL-1, IL-6 and IL-8, TNF alpha, NF-kappaB, ETS2, PTGS2, PTX3, CXCL16, KYNU, and CLEC4E were similarly (>2-fold) upregulated by LPS and MPLA in both species.

**Conclusion:**

The genomic responses of peripheral blood to LPS and MPLA in sheep are quite similar to those observed in humans, supporting the use of the ovine model for translational studies that mimic human inflammatory diseases and the study of TLR-based immunomodulators.

## Introduction

Basic science research, particularly studies involving animal models to mimic human illnesses, is pivotal for the understanding of disease pathophysiology, prevention and treatment. The question many researchers must ask, though, is what animal model should be used to best mimic a certain disease condition. The pathophysiological responses to the same injury or stimulus may vary in different species depending on anatomical structure, cellular composition, neurohumoral responses and gene expression. For example, ruminants, particularly ovine models, might be not the best choice for studies involving gastroenterological, specifically gastric, diseases as their anatomical structure considerably differs from that of humans—ruminants have multiple stomachs [1]. Similarly, the use of rodent models should be avoided for studies elucidating the pathophysiology of airway changes, particularly those involving airway secretory function, as the rodent lacks (mice) or possesses much fewer (rats) airway glands [2].

Sepsis is an increasingly common disease process that is associated with multi-organ dysfunction, leading to the death of 20–50% of patients [3]. To date, there is no Food and Drug Administration (FDA)-approved specific therapy available to treat this malady. There have been nearly 150 clinical trials performed to modulate inflammatory responses in critically ill patients, and all of these trials have failed [4–7]. Although there can be many different reasons for the failures of those clinical trials, it is likely that pitfalls in animal species selection may have played a considerable role.

In our previous studies, we have demonstrated the pathophysiology of ovine sepsis induced by Gram-positive and–negative microorganisms [8, 9], which displays similar cardiovascular hemodynamic changes i.e., tachycardia, increased cardiac output, systemic vasodilation with decreased vascular resistance, and heart failure, and closely resembles human hyperdynamic sepsis [8–10]. Our studies also indicate that sheep show hemodynamic and inflammatory responses to lipopolysaccharide (LPS) infusion that closely mimic those observed in humans [11, 12]. LPS is a component of the Gram negative bacterial cell wall that is recognized by the immune system after binding to the toll-like receptor 4 (TLR4) complex [13, 14]. LPS infusion causes many of the hemodynamic and inflammatory responses that are typical of clinical sepsis and is, thus, considered to be an important experimental model of sepsis.

Monophosphoryl lipid A (MPLA) is an LPS analog that possesses many of the immunomodulatory properties of endotoxin but does not induce severe inflammation or physiologic dysfunction at clinically useful doses. As such, MPLA is approved by the FDA for use as a vaccine adjuvant and is currently employed in several clinically important vaccines [15, 16]. More recent studies show that MPLA can augment innate immune responses to bacterial infections and could serve as a useful immunomodulatory to enhance the host response to infection in high-risk populations [17, 18].

In the present study, we aimed to compare the genomic responses of ovine and human whole blood exposed to the same doses of the TLR4 agonists LPS and MPLA. The goal was to define similarities and differences in the genomic response among the species. Given the similarities in the *in vivo* physiological and inflammatory responses among humans and sheep, this assessment will broaden the comparison and provide further information regarding the suitability of the ovine model for the study of sepsis and TLR-based immunomodulation.

## Material & Methods

### Sample Preparation

After obtaining Institutional Review Board (Human Research Protection Program, Vanderbilt University Medical Center, Nashville, TN) approval and informed consent, venous blood was withdrawn from six healthy human adult volunteers. All participants signed an informed (written) consent along with a trained member of the study team. The consent procedure was fully outlined in the approved Institutional Review Board application. Venous blood was also obtained from six healthy adult female sheep (approximately 3 years old) after obtaining Institutional Animal Care and Use Committee (The University of Texas Medical Branch at Galveston) approval. Blood was drawn into lithium heparin containing tubes. Three mL of blood was incubated at 37°C for 90 minutes on gently rolling rocker with 30 μL of PBS, or lipopolysaccharide (LPS) (Sigma Chemical, St Louis, MO) or monophosphoryl lipid A (MPLA) (Sigma Chemical, St Louis, MO). The doses for LPS and MPLA were 1 ug/mL and 10 ug/mL, respectively. After 90-minute incubation, 2.5 mL of blood was transferred to Paxgene tubes and incubated for 8 hrs at room air on a rolling rocker. Samples were stored in the freezer at -20°C until shipped to GenUs for the genomic assay.

### Isolation of total RNA

Human and sheep Total RNA was isolated from whole blood preserved in PAXgene Blood RNA tubes. Quality and quantity of the Total RNA sample was assessed using an Agilent Bioanalyzer with the RNA6000 Nano Lab Chip (Agilent Technologies; Santa Clara, CA) [19].

### cRNA preparation

Labeled cRNA was prepared by linear amplification of the Poly(A)+ RNA population within the Total RNA sample. Briefly, Total RNA was reverse transcribed after priming with a DNA oligonucleotide containing the T7 RNA polymerase promoter 5’ to a d(T)24 sequence. After second-strand cDNA synthesis and purification of double-stranded cDNA, in vitro transcription was performed using T7 RNA polymerase. The quantity and quality of the cRNA was assayed by spectrophotometry and on the Agilent Bioanalyzer as indicated for Total RNA analysis [20].

### Gene expression microarray

Purified cRNA was fragmented to uniform size and applied to Agilent Sheep 8x15K or Agilent Human 8x60K v2 Gene Expression microarray (Agilent Technologies, Sheep Design ID 019921, Human design ID 039494) in hybridization buffer. Arrays were hybridized at 37°C for 18 hrs in a rotating incubator, washed, and scanned with a G2565 Microarray Scanner (Agilent Technologies) [21].

### Data analysis

Arrays were processed with Agilent Feature Extraction software and data was analyzed with GeneSpring GX software (Agilent Technologies). To compare individual expression values across arrays, raw intensity data from each gene was normalized to the 75^th^ percentile intensity of each array. Genes were further normalized to the subject-specific PBS sample. Genes with values greater than background intensity in all replicates of at least one condition were filtered for further analysis. Differentially expressed genes were identified with Paired T-test p-values < 0.05 and fold change >1.5 fold. The p-value and stringency were chosen based on recommendations from the MAQC study [22, 23]. Orthologues were identified as Sheep probe Gene IDs listed in the NCBI Human orthologue table.

## Results

### RNA Quality

**Fig 1:**
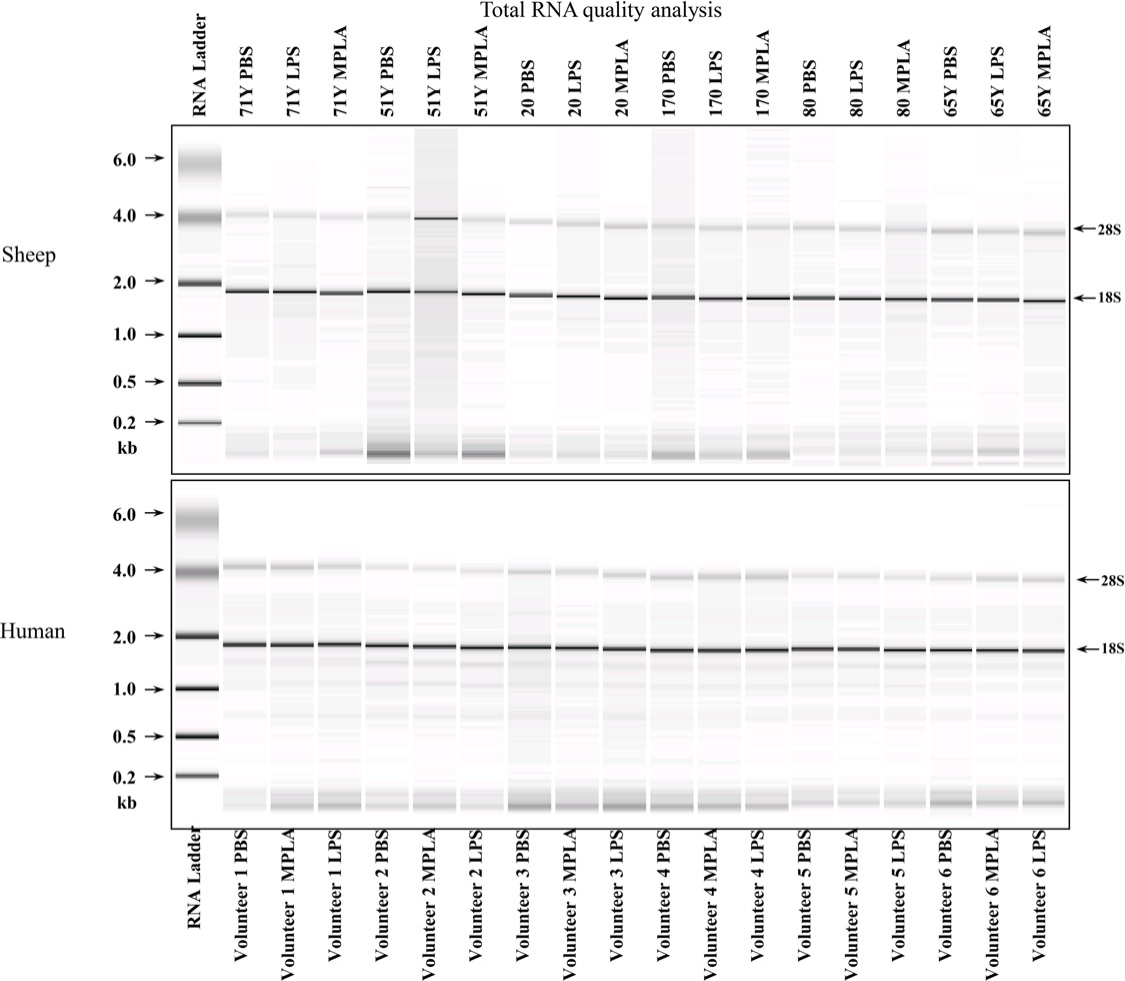
Total RNA quality analysis. Quality of the purified RNA was assessed by visualization of 18S and 28S RNA bands using an Agilent BioAnalyzer 2100 (Agilent Technologies, CA). Resulting electroperograms were used in the calculation of the 28S/18S ratio.

Quality of the purified RNA was assessed by visualization of 18S and 28S RNA bands using an Agilent BioAnalyzer 2100 (Agilent Technologies, CA). Resulting electroperograms were used in the calculation of the 28S/18S ratio and the RNA Integrity Number [19]. A 28S/18S ratio of 2 has been deemed acceptable RNA quality suitable for downstream applications. As evident in Fig 1, all RNA isolates resulted in very distinct 18S and 28S bands and thus deemed sufficient quality to proceed with expression analysis.

### Microarray fluorescence Intensity plots

**Fig 2:**
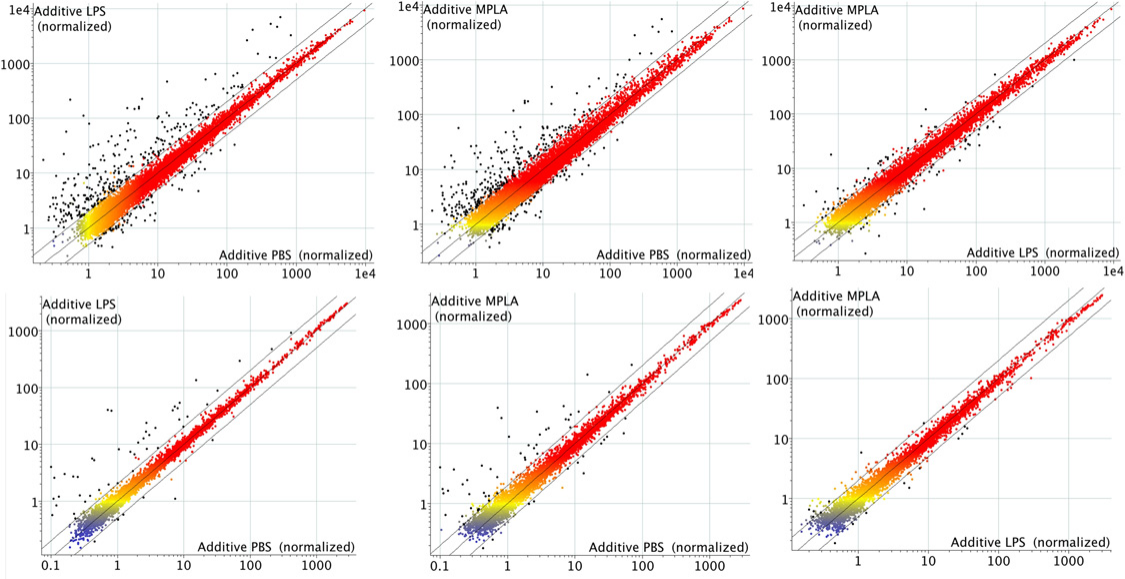
Scatter plots of probes above background in at least 4 of 6 replicates in any group (4,992 sheep probes, 11,431 human probes). Intensity values are normalized to the 75th percentile intensity of each array. Diagonal lines indicate 2-fold differential expression. Red/Orange: high expression; yellow: medium expression; blue: low expression; and black: probes that pass 2-fold and paired T-test p-value<0.05 cutoffs. Top row: Human. Bottom row: Sheep.

Normalized fluorescence intensity values were plotted for test samples versus the corresponding control’s values in scatter plots. Diagonal lines were drawn at the two-fold expression change threshold in order to clearly identify differentially expressed genes meeting that criterion. The 45-degree angle of these lines indicates effective normalization, as the majority of genes are unchanged in their expression. Furthermore, individual data points were colored black, denoting the change passed both the two-fold cutoff and a significant p-value of <0.05. LPS and MPLA exposure resulted in nearly equal numbers of genes being differentially expressed (Fig 2).

**Fig 3:**
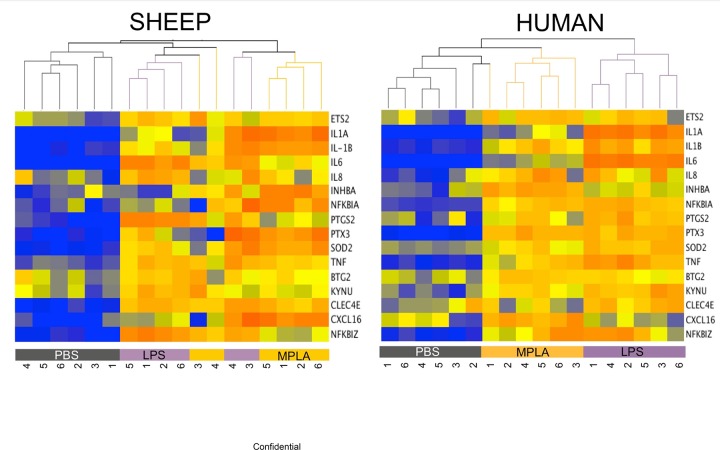
Hierarchical cluster of the 16 sheep-human orthologues (16 probes in both sheep and human) similarly expressed in both species (>1.5-fold, paired t-test p-values < 0.05). Intensity values are normalized to the mean expression within each subject. Red/Orange: high expression relative to median; yellow: mean expression; and blue: low expression relative to median.

In the Fig 3, we present a Hierarchical Clustering that was carried out on the subset of orthologous genes that exhibited greater than 1.5-fold induction and paired T-test value <0.05 after LPS or MPLA challenge in both species. LPS and MPLA are clearly discernable from PBS with pro-inflammatory cytokines being induced in both species with treatment. Not surprisingly, LPS is marginally discernable from MPLA.

### Venn diagram of differentially expressed genes in sheep

**Fig 4:**
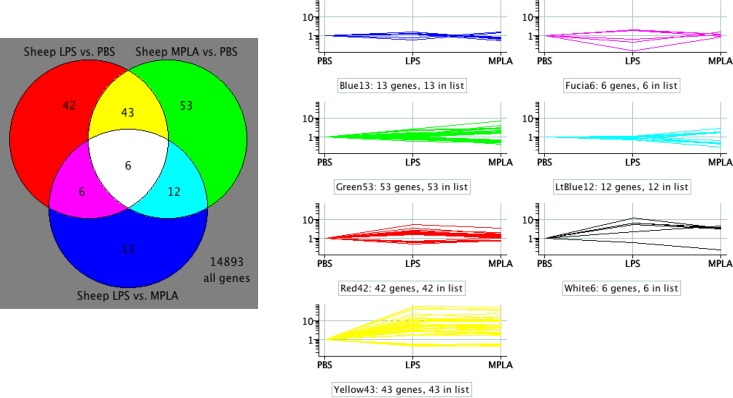
Differentially expressed sheep genes in at least one of three comparisons (>1.5-fold, paired t-test p-values <0.05, 175 probes) are displayed as normalized to the PBS sample within each individual. Line graph colors correspond to the colors in the Venn diagram (white = black).

A Venn diagram was generated in order to identify those genes that were uniquely induced by each treatment as well as those in common (Fig 4). Each circle of the Venn diagram contains genes differentially expressed > 1.5Fold and p-value < 0.05. Forty-three (yellow) of the differentially expressed genes exhibit expression changes common to both treatments whereas 42 were specific to LPS and 53 to MPLA. The line graph reveals the relative expression levels of the common and differentially expressed genes (Fig 4).

### Venn diagram of differentially expressed genes in human

**Fig 5:**
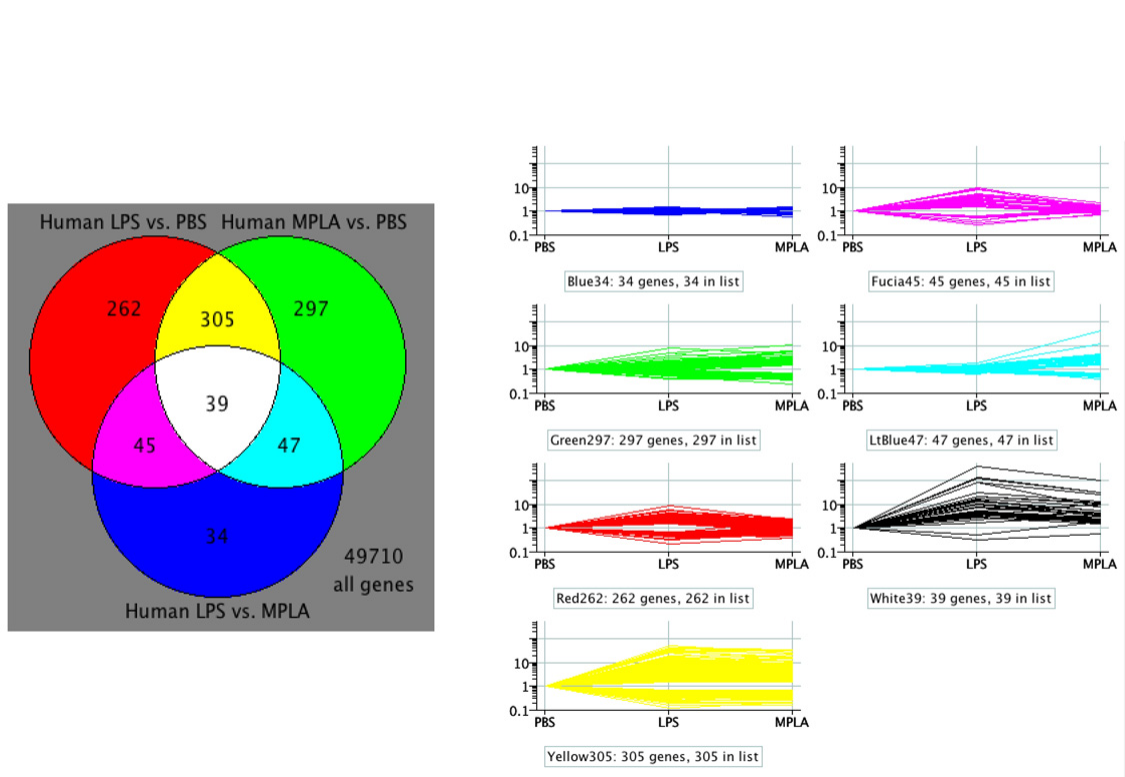
Differentially expressed human genes in at least one of three comparisons (>1.5-fold, paired t-test p-values <0.05, 1,029 probes) are displayed as normalized to the PBS sample within each individual. Line graph colors correspond to the colors in the Venn diagram (white = black).

A Venn diagram was generated for the human gene list resulting in 305 (yellow) of the >1.5-fold differentially expressed genes resulted in expression changes common to both treatments (Fig 5). Two hundred and sixty two were specific to LPS and 297 to MPLA. The line graph reveals the relative expression levels of the common and differentially expressed genes.

### The list of differentially (1.5-fold) expressed genes in both human and sheep

Tables 1 & 2 list orthologous genes for both sheep (Table 1) and human (Table 2), meeting the criteria of a >1.5 fold change in expression and a paired T-test p-value of <0.05 in at least one comparison. Of the 174 sheep probes with >1.5-fold change, 54 have an identified human orthologue. Twenty-two of the 54 orthologues show differential expression in the human samples. Although one does not expect identical fold changes when comparing two model systems, the direction and magnitude of expression changes in both sheep and human models strongly correlate.

**Table 1:** Orthologues that changed greater than 1.5-fold in sheep.

Gene Symbol	Sheep Blood						Probe ID
	LPS/PBS		MPLA/PBS		MPLA/LPS		
	Mean ratio	P-value	Mean ratio	P-value	Mean ratio	P-value	
BTG2	1.56	1.28E-02	1.36	2.16E-01	0.87	5.97E-01	A_70_P047196
CLEC4E	4.88	2.87E-05	4.77	2.88E-04	0.98	9.18E-01	A_70_P003521
CLEC4E	5.33	5.41E-05	4.64	1.03E-04	0.87	4.86E-01	A_70_P003522
CXCL16	4.70	6.39E-03	5.40	2.03E-02	1.15	8.25E-01	A_70_P057176
ETS2	1.80	3.50E-03	1.95	1.64E-02	1.08	7.22E-01	A_70_P049396
IL-1B	5.85	1.89E-03	6.21	2.98E-03	1.06	8.97E-01	A_70_P039761
IL-1A	19.19	9.08E-05	23.59	1.19E-04	1.23	6.16E-01	A_70_P051176
IL-1A	9.31	4.62E-04	12.71	7.57E-04	1.37	5.00E-01	A_70_P051177
IL-6	23.08	3.85E-04	13.43	1.28E-03	0.58	3.50E-01	A_70_P039706
IL-8	2.26	1.40E-02	1.52	1.81E-01	0.67	2.77E-01	A_70_P039662
IL-8	2.29	3.35E-02	1.42	2.53E-01	0.62	2.54E-01	A_70_P039661
INHBA	1.08	7.96E-01	2.93	1.58E-02	2.70	3.94E-02	A_70_P051056
INHBA	1.34	3.97E-01	2.95	4.22E-03	2.21	6.74E-02	A_70_P051057
KYNU	1.66	1.84E-02	1.53	1.15E-01	0.92	7.61E-01	A_70_P048371
NFKBIA	2.10	7.37E-02	2.61	1.87E-02	1.24	6.33E-01	A_70_P068111
NFKBIZ	6.35	2.46E-05	3.58	5.99E-04	0.57	2.35E-02	A_70_P018791
PTGS2	6.42	6.67E-04	3.51	7.33E-03	0.55	1.48E-01	A_70_P039256
PTX3	7.05	1.60E-03	5.44	3.34E-02	0.77	7.06E-01	A_70_P010836
PTX3	5.47	3.50E-03	3.51	7.55E-02	0.64	5.15E-01	A_70_P010837
SOD2	5.31	1.83E-04	4.63	7.64E-04	0.87	6.23E-01	A_70_P067986
SOD2	4.74	1.59E-04	4.46	3.92E-04	0.94	8.04E-01	A_70_P067987
TNF	2.79	3.06E-04	2.29	1.47E-04	0.82	1.98E-01	A_70_P030271

**Table 2:** Orthologues that changed greater than 1.5-fold in human.

Gene Symbol	Human Blood						Probe ID
	LPS/PBS		MPLA/PBS		MPLA/LPS		
	Mean ratio	P-value	Mean ratio	P-value	Mean ratio	P-value	
BTG2	1.58	2.73E-02	1.80	1.12E-02	1.14	5.51E-01	Hs.519162
CLEC4E	1.93	3.62E-02	1.04	9.23E-01	0.54	1.80E-01	A_33_P3318509
CXCL16	1.00	9.99E-01	2.55	2.04E-02	2.55	6.06E-02	A_23_P38505
ETS2	1.92	1.06E-01	2.35	1.51E-02	1.22	6.35E-01	A_24_P314179
IL-1B	8.77	7.28E-05	6.11	2.78E-05	0.70	1.36E-01	A_23_P79518
IL-1A	122.60	1.37E-05	30.01	4.49E-04	0.25	2.16E-02	A_23_P72096
IL-6	405.70	1.47E-05	105.00	9.17E-05	0.26	3.67E-02	A_23_P71037
IL-8	4.50	9.93E-03	4.35	3.20E-02	0.97	9.57E-01	A_32_P87013
INHBA	1.90	7.58E-02	3.70	3.57E-03	1.94	1.15E-01	A_23_P122924
KYNU	3.64	4.51E-03	2.68	3.01E-03	0.74	3.70E-01	A_24_P11506
NFKBIA	5.40	2.66E-06	4.75	7.06E-06	0.88	2.66E-01	A_23_P106002
NFKBIZ	10.28	1.08E-03	9.61	5.68E-04	0.94	8.85E-01	A_23_P212089
PTGS2	3.52	1.96E-02	2.38	9.20E-02	0.68	5.01E-01	A_24_P250922
PTX3	30.90	5.83E-05	30.75	8.73E-05	1.00	9.91E-01	A_23_P121064
SOD2	2.50	2.29E-05	1.82	1.77E-03	0.73	2.62E-02	A_23_P134176
TNF	10.48	1.19E-04	6.66	5.90E-05	0.64	1.29E-01	A_23_P376488

## Discussion

The main finding of the study was that LPS and MPLA provoked comparable gene expression responses in both human and sheep blood, inducing similar increases (>1.5–2fold) in gene products that serve as markers for inflammation and sepsis. Those include genes for IL-1, IL-6 and IL-8, TNF alpha, and NF-kappaB. We also found similar increases (in both species) of genes, such as ETS2, PTGS2, PTX3, CXCL16, KYNU, and CLEC4E that are actively involved or upregulated in inflammation. For instance, ETS2 is a transcriptional factor that mediates Rho-GEF Trio-induced pro-inflammatory downstream mechanisms [24]; PTGS2, known as cyclooxygenase-2, has been shown to be critically involved in pathophysiology of sepsis [25]; PTX3 levels are markedly increased in plasma (up to 1,500 ng/mL) during endotoxic shock, sepsis, and other inflammatory and infectious conditions, including *A*. *fumigatus* infection, meningococcal diseases, dengue, tuberculosis, and leptospirosis [26–30]; and CXCL16 is an inflammatory chemokine that serves as a marker of inflammation, atherosclerosis, and acute coronary syndromes in humans [31].

In the present study, we profiled 11,431 human and 4,992 sheep probes. We found 1,029 human and 175 sheep genes that were differentially expressed with more than 1.5-fold changes (p<0.05). Out of 175 sheep genes, 54 had a known human orthologue. Among the genes that have a human orthologue, 22 had > 1.5-fold changes in human samples, the majority of those changes were similar in direction and magnitude when comparing sheep to human blood. Although this study did not aim to individually describe all genes that were significantly altered by the stimulants (LPS and MPLA), the data strongly suggest that sheep genomic responses to LPS and MPLA are quite similar to those in human’s.

As mentioned, sepsis is an increasingly common malady in humans with mortality rates ranging from 20–50% depending on hospital site and the severity of sepsis [32, 33]. Despite recent advances in resuscitation and early antibiotic administration, the mortality rate for sepsis remains high [34]. Currently, there are no FDA-approved drugs for the treatment of sepsis despite a multitude of clinical trials. Some investigators have attributed the failure of past clinical trials to suboptimal animal models. The mouse has been the most commonly used animal model for the study of sepsis. A large number of interventions have shown promise in treating mouse sepsis but have not translated into clinical practice. Thus, recent papers have raised questions regarding the value of mouse models for the study of sepsis. Seok and colleagues reported that the whole blood transcriptional responses to LPS in mice were markedly different to that in humans [35]. Other investigators have pointed out differences in gene expression regulation and immunological phenotype among humans and mice [36, 37]. In addition to those biological differences, authors have pointed to modeling discrepancies that may attribute to suboptimal translation of results from the mouse to human [38]. For example, most murine studies employ inbred mice of young age and a single gender. Human populations are outbred and most patients that develop sepsis are older than the age of 65 years. Differences in the response to sepsis among males and females have also been demonstrated. Nevertheless, mouse models have been pivotal in defining innate and adaptive immune responses and have laid the groundwork to understanding biological responses to infection. Osuchowski and colleagues [39] have pointed out that murine models mimic the human response to sepsis in many ways at the physiologic and immunologic levels. Thus, evolution of mouse models for sepsis research to those that more closely mimic human demographics and characteristics may improve future translational research.

Given the current controversies surrounding mouse models of sepsis, it is important to consider alternative animal models to advance treatment of this common and debilitating condition. The ovine model has many attractive characteristics. The present study demonstrates similarities in the whole blood transcriptional response among humans and sheep. Past studies have also demonstrated that physiological responses to sepsis in the ovine model mimic those of human sepsis.

Ovine sepsis models have been well characterized and used for years in our Large Animal Translational Intensive Care Unit as well as in other large animal research facilities. The ovine endotoxemia model was developed in our laboratory, and the cardiopulmonary responses were extensively studied [40–43]. These studies showed that continuous infusion of very low doses (9, 12 and 24 ng/kg/h) of LPS closely resembled the hyperdynamic sepsis observed in septic patients and showed that sheep exhibit a sensitivity to LPS that is very similar to humans [41]. The sheep displayed increased cardiac output, decreased systemic vascular resistance and increased pulmonary artery pressure after the LPS administration [41]. The pulmonary lymph flow was dose-dependently increased by LPS and associated with a transient leukocytopenia, which was followed by leukocytosis starting at 4 hours after LPS administration. The results of these early studies with intravenously administered LPS indicate that sheep responses to LPS challenge are quite similar to a human’s, at least in regard to cardiopulmonary hemodynamics and in dose-dependent sensitivity. Compared to the mouse model, the sensitivity of sheep to LPS challenge is attractive since mice are resistant to LPS challenge and require doses of several orders of magnitude higher than in humans to induce similar pathophysiology.

Previous studies have also demonstrated anatomical, physiological and genomic comparability among sheep and humans during sepsis. Gajewski and colleagues showed no differences between human and sheep coagulation factors, such as activated partial thromboplastin time (aPTT) or prothrombin (PT) or factors II, V, VII, X [44]. Although factor VIII was slightly higher in ovine blood than in humans, these findings indicate quite a comparable pattern in coagulation system activation between the two species [44]. Craig and colleagues [45] reported that the "thromboangiitis" seen in non-pulmonary sepsis in sheep is almost identical to the vascular lesions described in human ARDS. Sheep have airway structures and functions i.e., airflow, resistance, compliance, respiratory rate and tidal volumes, similar to humans [46]. They have tonsils, bronchial glands, mast cell distributions, sensory nerves, airway capillaries and cartilage distributions, and mucus secreting cells [46] analogous to humans and the inhalation of allergens induces similar allergic reactions characterized by both early and late phase bronchoconstrictive responses and airway hyper-responsiveness [47, 48].

In addition to assessing the genomic response to LPS, we compared the genomic response of sheep and human blood to MPLA. MPLA is a TLR4 agonist and lipid A mimetic that is created by removal of the C1 phosphate group from native lipid A by hydrolysis or by *de novo* synthesis [18, 49]. That alteration decreases the pro-inflammatory effects of MPLA by greater than 100-fold compared to native lipid A [50]. Yet, MPLA retains potent immunomodulatory properties and is currently employed as an adjuvant in FDA-approved papillomavirus and hepatitis B vaccines and is under investigation for use in numerous other vaccine systems [15, 51]. Additional research shows that treatment with MPLA will augment the innate host response to bacterial infection [52]. However, the whole blood genomic response to MPLA has not been previously characterized. Our results show that MPLA induces less robust transcription of pro-inflammatory genes such as IL-1α, IL-1β, IL-6 and IL-8 compared to LPS in both species. We also observed that MPLA induces a unique transcriptional profile compared to LPS that is characterized by induction of 53 and 297 unique genes in sheep and humans, respectively, compared to LPS. These findings confirm the lesser pro-inflammatory profile of MPLA compared to LPS and its ability to uniquely modulate leukocyte immune responses. Our study also shows a similar genomic response to MPLA among sheep and humans, which supports the use of sheep for pre-clinical studies of MPLA.

The primary limitation of this study was that it compared the transcriptional response to MPLA and LPS in isolated human and sheep blood, but not under *in vivo* conditions. While the current study provides a useful comparison, future studies using blood taken from septic humans and sheep will serve to broaden these initial observations. However, it is worth noting that the comparison of genomic responses in human vs. ovine sepsis models are quite complicated because of challenges of matching causative infectious agents, concomitant morbidities, sepsis duration, severity of multiple organ failure, therapeutic interventions and other variables. The focus of the study on blood genomic responses also provides some limitation since genomic responses in other tissues such as lung, kidney and liver may vary from the changes noted in the blood. Nevertheless, blood is easily accessible and provides the only available tissue that can be routinely harvested from humans under most conditions.

In summary, the results of our present study indicate that the genomic responses to LPS and MPLA in sheep blood are quite similar to those observed in human blood. Based on these findings and previous pathophysiologic studies, the ovine model of sepsis provides an attractive model for translational research in the field of sepsis and the study of TLR-based immunomodulators such as MPLA.
